# The impact of Susan G. Komen-funded research on approved drugs for breast cancer treatment

**DOI:** 10.1017/cts.2025.10224

**Published:** 2025-12-12

**Authors:** Dana M. Brantley-Sieders, Lauren Leslie, Kimberly Sabelko, Amy M. Dworkin, Kari Wojtanik

**Affiliations:** 1 Research Evaluation Manager, Applied Research & Evaluation, Susan G. Komen, Dallas, TX, USA; 2 Research Program Evaluator, Applied Research & Evaluation, Susan G. Komen, Dallas, TX, USA; 3 Vice President of Scientific Strategy & Programs, Susan G. Komen, Dallas, TX, USA; 4 Director, Research Evaluation, Applied Research & Evaluation, Susan G. Komen, Dallas, TX, USA; 5 Senior Director, Applied Research & Evaluation, Susan G. Komen, Dallas, TX, USA

**Keywords:** Breast cancer, Susan G. Komen, research funding, breast cancer drugs, research impact assessment

## Abstract

**Introduction:**

Susan G. Komen® (Komen) has invested nearly $1.1 billion in ground-breaking breast cancer research since 1982. As a patient-centered organization, Komen measures research funding impact beyond academic metrics in favor of economic value and societal effects. Here, we highlight an innovative approach to assessing the real-world impact of Komen-funded research by showing how Komen funding for pivotal studies and key personnel conducting research contributed to discovery and development of targeted therapy drugs approved to treat breast cancer by the United States Food and Drug Administration (FDA) between 2012 and 2023.

**Methods:**

We utilized bibliometric analysis to work backwards through citations within pivotal Phase III clinical trial publications to identify earlier clinical trials, pre-clinical research, and basic research publications that supported development of each drug, evaluating each published study and contributing authors for Komen funding support.

**Results:**

All 19 targeted therapy drugs approved by the FDA between 2012 and 2023 were impacted at multiple phases along the drug development pipeline by Komen funding, including direct impacts in basic research (i.e., investments in projects) that supported target discovery, and impacts in support of key personnel who contributed to pivotal studies and clinical trials (i.e., investments in people) that led to approval.

**Conclusions:**

Nonprofit and public sector research funding provide the foundation for the drug development pipeline. This paper highlights an innovative approach to assess the impact of research investments beyond traditional academic measures and underscores the significance of nonprofit, patient-centered organizations like Komen in driving drug development through supporting basic and applied research.

## Introduction

Susan G. Komen’s® (Komen) mission is to save lives by meeting the most critical needs in our communities and investing in breakthrough research to prevent and cure breast cancer [1]. Komen has invested nearly $1.1 billion in over 2,800 research grants since 1982 [2]. As a patient advocacy organization, Komen understands that research is one of our best weapons against breast cancer, and supporting innovative research brings new treatment paradigms and practice changes that benefit patients. We are committed to demonstrating the long-term, real-world impacts of Komen’s research investments, especially as they relate to new treatments and improvements in patient care and outcomes. The development of new targeted therapy drugs that advance precision medicine is a critical part of this commitment.

The drug development pipeline from basic and pre-clinical research to clinical trials that support FDA approval/commercialization [3,4] is lengthy and expensive, averaging 10–15 years and $3 billion or more, with investments by multiple funders and involving hundreds of researchers and thousands of patients who participate in clinical trial studies. [5–7]. While challenging, Komen continually seeks to understand how its specific research investments promote progress across the drug development pipeline. To tackle this challenge, Komen develops and uses innovative Research Impact Assessment (RIA) [8] strategies to more accurately and comprehensively measure the impact of its research funding. Using the Payback Framework [9] as an RIA guide, Komen evaluates how its funded research translates into the development and use of “products” with tangible “health and health sector benefits.” This approach provides a more accurate assessment of the ways research funding ultimately improves clinical care and outcomes for people with breast cancer relative to traditional academic metrics often used to evaluate research impact (e.g., citations/publications, career progression of funded investigators, acquisition of follow-on funding after the initial grant, classified as “knowledge production”) in the Payback framework [9].

One tool Komen developed involves tracking potential products that could result from each funded grant. Using a novel Komen Product Tracking System, each funded grant is classified by product potential (e.g., treatment, biomarker, behavioral intervention, healthcare tool that improves access to care and/or alleviates disparities in care, etc.) and by stage in the research pipeline (e.g., basic research, pre-clinical research, clinical trials, and regulatory approval/commercialization). Progress through the research pipeline is monitored during the funding period and beyond [10].

In this study, we used another innovative RIA strategy to retrospectively analyze published scientific literature to determine how Komen’s research funding supported discovery, pre-clinical development, and clinical testing of drugs that have revolutionized breast cancer treatment, actively benefiting society and the communities Komen serves. We show that Komen research funding supported the discovery and development of all 19 targeted therapy drugs FDA approved to treat breast cancer between 2012 and 2023. All drugs were impacted at multiple phases along the drug development pipeline, including several direct impacts in basic research (i.e., investments in projects) that supported target discovery, and impacts in support of many key personnel who contributed to pivotal studies that led to approval for these drugs in clinical trials (i.e., investments in people).

## Materials and methods

### Evaluation strategy

Evaluation of Komen’s research funding impact followed the Payback Framework originally developed by Martin Buxton and Stephen Hanney [9], a research tool to classify the individual “paybacks” from investments in research, such as knowledge production, informing policy and product development, health sector benefits, and broader economic benefits. This evaluation focused on assessing payback or return on investment in the “product development” and “health/health sector benefits” areas of the model, specifically, the development of FDA approved drugs.

### Drug identification

Data sourced from the FDA [11] and the United States National Cancer Institute [12] websites identified 19 targeted therapy drugs that were FDA approved for breast cancer treatment between 2012 and 2023. The compound name, trade name, drug class, indication(s) for first and expanded FDA approvals, and years for all FDA approvals in breast cancer treatment were collected.

### Bibliometric linkage strategy

Publications that led to first and expanded FDA approval (i.e., pivotal Phase III clinical trial study publications) for each drug were identified from prescribing information and validated by press releases and data curated by https://www.drugs.com/ and https://clinicaltrials.gov/. Author information and citations within these publications were extracted, and bibliometric linkage analysis performed using the Dimensions® for Funders platform Application Programming Interface (API) technology, allowing us to work backwards through the cited literature to identify associated pivotal study publications for Phase II and Phase I clinical trials, pre-clinical studies, basic research studies, and associated authors. Review papers cited by these publications were used to validate relevant pivotal studies and to fill in gaps.

### Inclusion and exclusion criteria

Publications were included if studies were English language and were full-text research articles, reports, or published meeting abstracts. Publications reporting clinical trials for drugs in the same class or comparisons with standard of care drugs used to treat the same breast cancer subtypes were included, as these studies were considered foundational to development and efficacy assessment of the evaluated drug. We manually excluded review publications and publications describing: (1) previous clinical practice guidelines; (2) widely used scientific methods; (3) publications describing commonly used cell lines and *in vivo* models that were not novel/created in the process of target validation; (4) letters to editors; and (5) prescribing information for existing drugs. Deduplication was performed using the API.

### Bibliometric analysis

Pivotal studies were identified in an unbiased manner. Pivotal publications were collected and author lists extracted for bibliometric linkage analysis using Dimensions® API. We evaluated each published study and contributing authors for Komen support by cross-referencing Komen’s database of supported grants and key personnel. Key personnel were categorized as principal investigator (PI) and/or all other named personnel within a study, including: co-investigator, collaborator, consultant, co-PI, graduate student/trainee, postdoctoral or medical fellow, mentor, and patient advocate.

### Evaluating Komen research funding impact

To define the links between Komen funding and each drug, we identified specific “touchpoints” for pivotal studies including Komen funding for the published study project (i.e., direct touchpoints) and/or Komen support of key personnel who contributed to the pivotal study research (i.e., investigator touchpoints). Direct touchpoints were confirmed by reviewing the acknowledgements sections of publications, reviewing Dimensions® for links to Komen funding, and/or inclusion of study publications in progress reports submitted to Komen by funded investigators when available. Investigator touchpoints were defined by funding for investigators prior to or overlapping with the pivotal study publication year or clinical trial period. Each investigator touchpoint represents a specific author, and studies with multiple investigator touchpoints include a single touchpoint for each author regardless of the number of grants that preceded or overlapped with study period.

## Results

### Identifying targeted therapy drugs approved between 2012 and 2023

We identified 19 targeted therapy drugs first approved by the FDA [13] between 2012 and 2023 for breast cancer treatment (Table 1). Ten drugs also received expanded use approval for early-stage breast cancer after initial approval for treatment of metastatic disease (Table 2). These drugs are used to treat many breast cancer subtypes, including HER2-positive [14], triple negative [15], hormone receptor-positive [14], and hereditary breast cancers driven by germline mutations in Breast Cancer genes 1 and 2 (*BRCA*1 and *BRCA*2) [16]. Drug classes include kinase inhibitors, antibody and antibody drug conjugates, immune checkpoint inhibitors, and PARP inhibitors.

**Table 1.** Nineteen drugs first FDA approved to treat breast cancer between 2012 and 2023

Drug	Breast cancer subtype	Year first FDA approved	Drug class	Indication first approval^a^
Everolimus	HR+	2012	mTORi	MBC [34]
Pertuzumab	HER2+	2012	Antibody	MBC [35]
Ado-trastuzumab emtansine	HER2+	2013	ADC	MBC [36]
Palbociclib	HR+	2015	CDK4/6i	MBC [37]
Abemaciclib	HR+	2017	CDK4/6i	MBC [17]
Ribociclib	HR+	2017	CDK4/6i	MBC [38]
Neratinib	HER2+	2017	HER/EGFRi	Early BC [39]
Olaparib	gm*BRCA*	2018	PARPi	MBC [40]
Talazoparib	gm*BRCA*	2018	PARPi	MBC [41]
Alpelisib	HR+	2019	PI3Ki	MBC [42]
Fam-trastuzumab deruxtecan	HER2+	2019	ADC	MBC [43]
Trastuzumab hyaluronidase	HER2+	2019	Antibody	Early BC and MBC [44]
Pertuzumab/trastuzumab & hyaluronidase	HER2+	2020	Antibody	Early BC and MBC [45]
Margetuximab	HER2+	2020	Antibody	MBC [46]
Tucatinib	HER2+	2020	HER2i	MBC [47]
Sacituzumab govitecan	TNBC	2020	ADC	MBC [48]
Pembrolizumab	TNBC	2020	ICI	MBC [49]
Elacestrant	HR+	2023	SERD	MBC [50]
Capivasertib	HR+	2023	AKTi	MBC [51]

HER2/HER2-positive breast cancers (HER2+), triple negative breast cancers (TNBC), hereditary breast cancers driven by germline mutations in *BRCA* genes 1 or 2 (*gmBRCA*), hormone receptor-positive breast cancers (HR+), metastatic breast cancer (MBC) or early breast cancer (BC), mTOR inhibitor (mTORi), antibody drug conjugate (ADC), CDK4/6 inhibitor (CDK4/6i), HER2/EGFR inhibitor (HER2/EGFRi). PARP inhibitor (PARPi), PI3K inhibitor (PI3Ki), HER2 inhibitor (HER2i), immune checkpoint inhibitor (ICI), selective estrogen receptor degrader (SERD), Akt inhibitor (AKTi).

a FDA approval for use in earlier stages has since been expanded for several of these drugs (Table 2).

**Table 2.** Ten drugs that received one or two expanded FDA approvals to treat breast cancer between 2012 and 2023

Drug	Year expanded FDA approval 1	Drug class	Indication expanded FDA approval 1	Year expanded FDA approval 2	Indication expanded FDA approval 2
Pertuzumab	2013	Antibody	Neoadjuvant, HER2+ BC [35]	2017	Adjuvant early stage HER2+ BC [35]
Ado-trastuzumab emtansine	2019	ADC	Adjuvant early stage HER2+ BC [36]		
Palbociclib	2016	CDK4/6i	HR + MBC; combined with fulvestrant [37]	2019	HR + MBC; combined with aromatase inhibitor [37]
Abemaciclib	2018	CDK4/6i	HR + MBC; combined with aromatase inhibitor [17]	2021	Adjuvant early stage HR + BC; high risk for recurrence [17]
Ribociclib	2018	CDK4/6i	Pre-and peri-menopausal HR + BC [38]	2021	Male HR + BC [38]
Neratinib	2020	HER2/EGFRi	HER2+ MBC [39]		
Fam-trastuzumab deruxtecan	2022	ADC	HER2+ MBC; with prior anti-HER2 treatment [43]	2022	HER2-low MBC [43]
Olaparib	2022	PARPi	Adjuvant early stage gm*BRCA* BC [40]		
Sacituzumab govitecan	2021	ADC	First line treatment Metastatic TNBC [48]	2023	Pre-treated metastatic TNBC [48]
Pembrolizumab	2021	ICI	High-risk early stage TNBC [49]		

Antibody drug conjugate (ADC), CDK4/6 inhibitor (CDK4/6i), HER2/EGFR inhibitor (HER2/EGFRi). PARP inhibitor (PARPi), immune checkpoint inhibitor (ICI), HER2/HER2-positive breast cancers (HER2+), triple negative breast cancers (TNBC), hereditary breast cancers driven by germline mutations in *BRCA* genes 1 or 2 (*gmBRCA*), hormone receptor-positive breast cancers (HR+), metastatic breast cancer (MBC) or early breast cancer (BC).

### Identifying pivotal studies in the drug development pipeline

**Figure 1.**
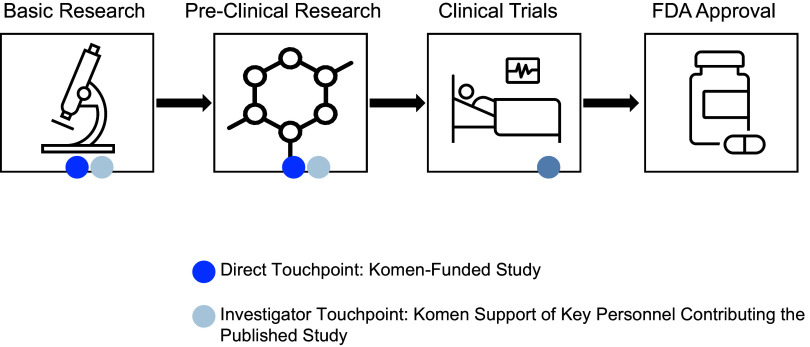
Identifying Komen Touchpoints Across the Drug Development Pipeline. The drug development pipeline that brings drugs from the laboratory to the clinic involves basic research, pre-clinical research, clinical research in the form of clinical trials, and approval for clinical use and commercialization. Our goal was to identify Komen touchpoints, including Komen research funding for studies (direct touchpoints) and Komen research funding for key personnel who contributed to pivotal studies (investigator touchpoints) in the first three phases of the translational spectrum for each drug that contributed to eventual FDA approval.

**Figure 2.**
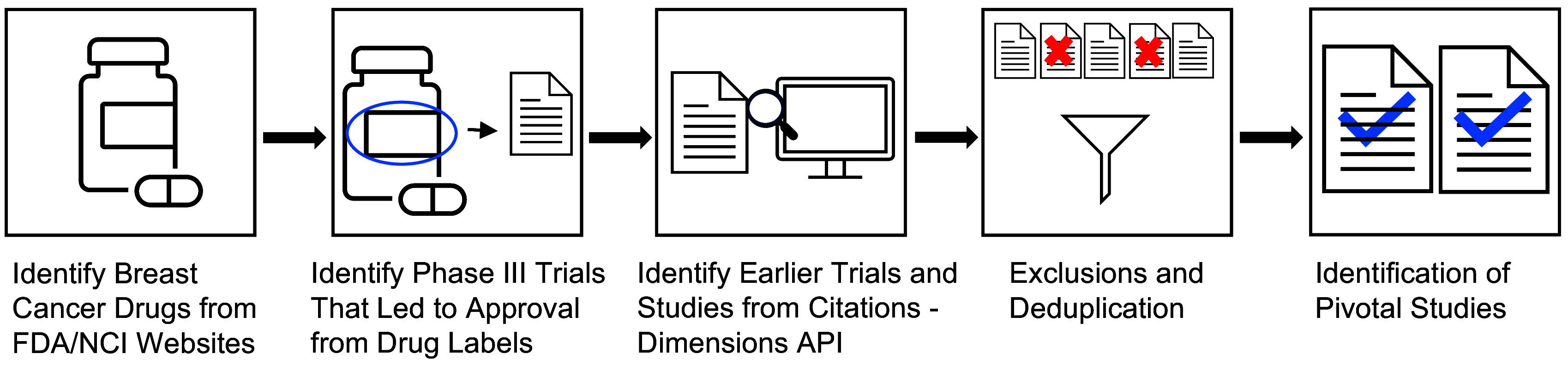
Strategy for Identifying Pivotal Studies Across the Drug Development Pipeline. 19 drugs FDA approved to treat breast cancer between 2012 and 2023 were identified. Next, pivotal Phase III trials were identified from prescribing information labels and press releases. Bibliometric linkage analysis of citations from pivotal Phase III clinical trial study publications was performed using Application Programming Interface (API) technology in the Dimensions® for Funders platform, working backwards to capture pivotal Phase II and Phase I clinical trial studies, pre-clinical studies, and basic research studies. The process was repeated for citation results from Phase II and Phase I study publications and pre-clinical research publications, de-duplicating using the API. Certain types of publications were manually excluded. This process identified pivotal study publications that led to FDA approval of 19 drugs between 2012 and 2023.

Extensive research was conducted along the drug development pipeline that led to FDA approval and commercialization for these drugs (Figure 1) [3]. Points of impact for Komen research funding, or “touchpoints,” across the pipeline were identified in the pivotal studies that supported first and expanded FDA approval. Touchpoints included Komen funding for the published study project (direct touchpoints), and/or Komen support of key personnel contributing to the published study (investigator touchpoints; Figure 1). Using citation linkage analysis described in the Methods, we identified connections between the pivotal Phase III clinical trial publications and previous pivotal studies supporting this work from Phase II and Phase I clinical trials, pre-clinical studies, and basic research studies (Figure 2).

We identified and reviewed 844 pivotal publications across all phases in the pipeline related to first FDA approval for all 19 drugs, identifying 20 to 65 publications per drug (average 44 publications per drug). For example, we identified 35 pivotal study publications across the pipeline for the drug abemaciclib published between 1989 and 2017 that supported first FDA approval [17]. Following first FDA approval [13], drugs can be tested for new indications (e.g., treatment for earlier disease stages or in new combinations) and receive expanded approval. Of the 19 approved drugs, 10 received one or more expanded FDA approvals for breast cancer treatment between 2012 and 2023 (Table 2). We identified 54 pivotal publications supporting expanded approvals for these 10 drugs, with 2 to 11 publications per drug (average of 5 publications per drug).

### Komen funding contributed to the development of all 19 drugs approved by the FDA between 2012 and 2023 to treat breast cancer

Since 1982, Komen has funded over 2,800 research grants supporting more than 5,000 key personnel [18]. We first assessed pivotal study publications identified for each drug for direct Komen research funding support. Each pivotal study publication had between 2 and 50 contributing authors. To identify investigator touchpoints, we extracted author information from each pivotal study identified for each drug and cross-referenced the author lists with Komen’s database of supported grants and key personnel.

**Figure 3.**
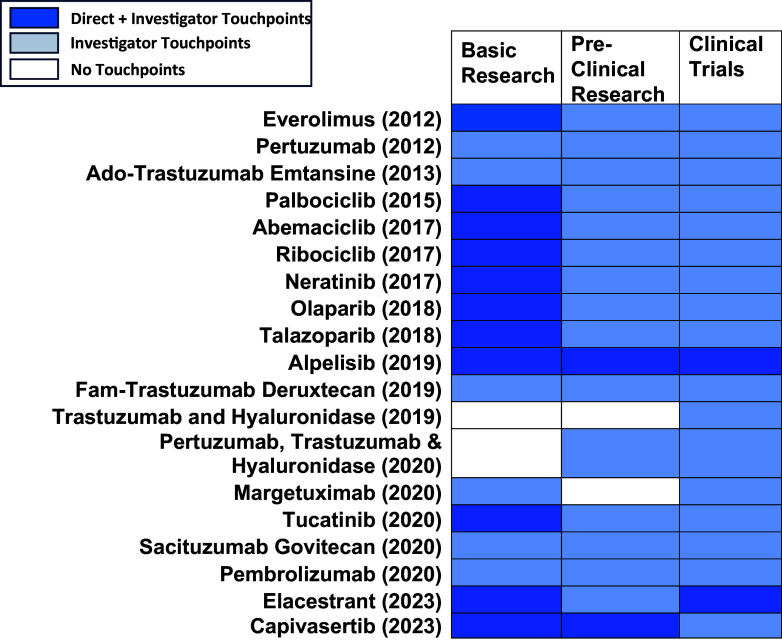
Touchpoints for First FDA Approval of Drugs by Research Category. Drugs are listed by their generic (non-trade) names with date of first approval in parentheses. Komen touchpoints across phases of the drug development pipeline, including basic research, pre-clinical research, and clinical research (Phase I, Phase II, and Phase III clinical trials) are shown. Drugs that had both direct and investigator touchpoints in the research category are noted in dark blue. Drugs that had only investigator touchpoints in the research category are noted in light blue. White indicates that no touchpoints were found.

We identified 983 Komen touchpoints across the drug development pipeline for all 19 drugs at first approval, including direct and investigator touchpoints. Each drug had Komen touchpoints in at least one phase in the pipeline (Figure 3). All drugs had investigator touchpoints, and 58% (11 out of 19) of drugs had both direct and Komen investigator touchpoints. All 19 drugs had touchpoints in the clinical trials phase. We found touchpoints in 17 out of 19 drugs (89%) in basic research, as well as pre-clinical research.

When classifying the touchpoints for all drugs by pipeline phase, 64% of total touchpoints (combined direct and investigator touchpoints) were identified at the clinical trial phase (Phases I–III; Table 3). 23% of total touchpoints were identified in basic research, and 13% were identified in pre-clinical research (Table 3). This demonstrates the overall impact of Komen’s investment in pivotal studies and contributing investigators that supported development of all 19 targeted therapy drugs FDA approved to treat breast cancer between 2012 and 2023.

**Table 3.** Overall investigator and direct komen touchpoints for nineteen drugs first FDA approved to treat breast cancer between 2012 and 2023 by research phase

Overall touchpoints		
**Research phase**	**Number**	**Percentage**
Basic research	224	23%
Pre-clinical research	130	13%
Clinical trials	629	64%
**Total overall touchpoints**	983	
**Investigator touchpoints**		
**Research phase**	**Number**	**Percentage**
Basic research	177	19%
Pre-clinical research	123	13%
Clinical trials	626	68%
**Total investigator touchpoints**	926	
**Direct touchpoints**		
**Research phase**	**Number**	**Percentage**
Basic research	47	83%
Pre-clinical research	7	13%
Clinical trials	3	5%
**Total direct touchpoints**	57	

Komen touchpoint calculations for overall investigator and direct touchpoints broken down by research phase (basic research, pre-clinical research, clinical trials). Touchpoint numbers in each research phase are the sum of touchpoints for all 19 drugs in that category. Percentages are based on total touchpoints for overall investigator and direct touchpoints.

We also assessed which portions of the drug development pipeline had the greatest impacts from direct versus investigator touchpoints. Basic research studies had the highest number of direct touchpoints with 47 touchpoints across 11 of the 19 drugs (Table 3). This was followed by pre-clinical research (7 touchpoints) and clinical trials (3 touchpoints) with 1 drug, alpelisib, having direct touchpoints for each phase (Figure 3). Basic research studies are foundational in the drug development pipeline and encompass the discovery of new drug targets, their biological functions, and relevance to treating breast cancer. Thus, our findings highlight the importance of supporting basic research and demonstrate the impact of Komen’s direct research funding on this crucial phase of the pipeline.

Komen invests in the best and brightest investigators in breast cancer research, whose innovative work drives progression along the drug development pipeline. Thus, we sought to determine which portions of the drug development pipeline had the greatest impacts from investigator touchpoints that reflect Komen’s research funding impact through investments in people. 926 Komen investigator touchpoints were identified for all 19 targeted therapy drugs FDA approved to treat breast cancer between 2012 and 2023 in multiple phases of the drug development pipeline, with the greatest impact in clinical trials research (68%; Table 3; Figure 3). 19% of investigator touchpoints were identified in basic research and 13% in pre-clinical research (Table 3). Our findings reflect how ongoing research funding support enables investigators to contribute to translational breast cancer research that helps bring new treatments to the clinic.

**Figure 4.**
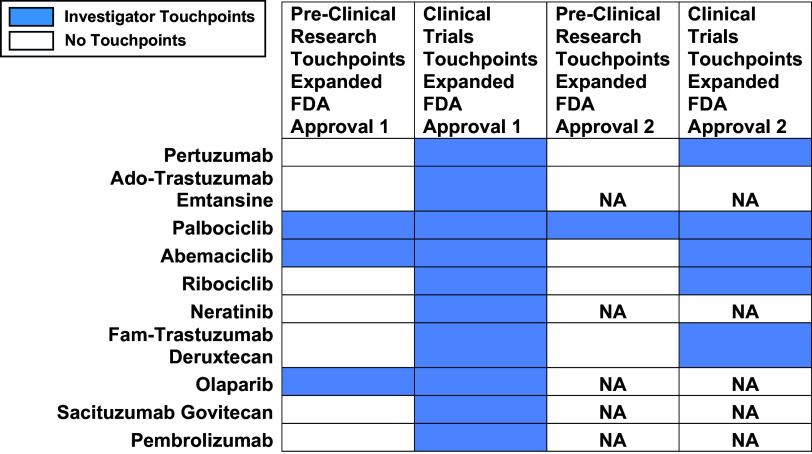
Touchpoints for Expanded FDA Approval of Drugs by Research Phase. Ten drugs that received expanded FDA approvals between 2012 and 2023 are listed by their generic (non-trade names). Five drugs received a second expanded approval within this timeframe. Komen investigator touchpoints across phases within the drug development pipeline, including pre-clinical research and clinical research (Phase I, Phase II, and Phase III clinical trials) are shown in light blue. White indicates that no touchpoints were found in the research category. NA indicates that the drug did not receive a second expanded approval within this timeframe. Few if any basic research studies were identified as fundamental studies for expanded drug approvals, and no Komen touchpoints were identified in these studies. No direct touchpoints were identified for expanded approvals.

Komen investigator touchpoints were also found for all 10 drugs that received a first expanded FDA approval, and for all 5 drugs that received a second expanded FDA approval (Figure 4). 2 drugs received a third expanded approval: ribociclib, with no Komen touchpoints; and abemaciclib, with more than 40 investigator touchpoints in the clinical trials phase. Most investigator touchpoints were found in the clinical trials phase, with 80% (144 out of 180) of touchpoints, followed by pre-clinical research with 20% (36 out of 180) of touchpoints. We found no investigator touchpoints in basic research, likely because fewer basic research studies were identified that supported expanded approvals.

## Discussion

To date, Komen has invested nearly $1.1 billion in breast cancer research, more than any other nonprofit outside of the United States government. It is important that Komen clearly demonstrate the value of its funded research so our partners and the communities we serve see how funded research translates into real-world advances in breast cancer clinical care and improved outcomes for people with breast cancer. Our evaluation of Komen’s research funding impact on the development of all 19 drugs FDA approved to treat breast cancer between 2012 and 2023 addresses this important impact measure of research. Though breast cancer incidence increased between 2012 and 2023, due in part to an aging population and growing proportion of cases in younger women, mortality rates declined and survival improved [19], with overall relative survival rates for women diagnosed with breast cancer increasing by 2%–3% at 5, 10, and 15 years between 2012 and 2023 [20,21]. While this is attributable, in part, to improved screening and diagnostic tools, the development and approval of new molecularly targeted therapies during this timeframe also likely played a major role, particularly in outcomes for metastatic breast cancer. The majority of the 19 new drugs developed within this timeframe were first approved to treat metastatic breast cancer, lengthening survival in clinical trials (Table 4). 5-year relative survival rates increased for MBC from 18% (2005–2012) to 32% (2015–2021) [22], further supporting the notion that the development of new drugs resulted in improved outcomes for people with breast cancer during this timeframe. Moreover, targeted therapy drugs like the 19 evaluated in this study cause less harm to normal cells than chemotherapy in general, which may mean fewer side effects [23]. Still, it is important to note that these drugs are also associated with side effects, such as gastrointestinal side effects, changes in skin, hair and nails, neutropenia, and lung and cardiovascular toxicities [24]. Moreover, financial toxicity, or the harmful effect of high cost of treatment on a person’s quality of life, is associated with many drugs used to treat cancer [25]. Komen and other funders are supporting research focused on approaches to mitigate adverse effects. In addition, Komen and other patient advocacy organizations have programs to help address financial and other barriers to care [26].

**Table 4.** Survival data from phase III clinical trials for nineteen drugs first FDA approved to treat breast cancer between 2012 and 2023

Drug	Breast cancer subtype	Phase III clinical trial (name, National Clinical Trial identifier)	Reported survival benefit first approval	Reported hazard ratio
Everolimus	HR+	BOLERO-2, NCT00863655	4.6 months PFS	0.45 [34]
Pertuzumab	HER2+	CLEOPATRA, NCT00567190	51 months PFS	0.62 [35]
Ado-trastuzumab emtansine	HER2+	EMELIA, NCT00829166	3.2 months PFS	0.65 [36]
Palbociclib	HR+	PALOMA-2, NCT01740427	10.3 months PFS	0.58 [37]
Abemaciclib	HR+	MONARCH-2, NCT02107703	7.1 months PFS	0.55 [17]
Ribociclib	HR+	MONALEESA-2, NCT01958021	12.5 months OS	0.77 [38]
Neratinib	HER2+	ExteNET, NCT00878709	2.3 months iDFS	0.66 [39]
Olaparib	gm*BRCA*	OlympiAD, NCT02000622	16 months PFS	0.53 [40]
Talazoparib	gm*BRCA*	EMBRACA, NCT01945775	3 months PFS	0.54 [41]
Alpelisib	HR+	SOLAR-1, NCT02437318	26 months PFS	0.65 [42]
Fam-trastuzumab deruxtecan	HER2+	DESTINY-Breast03, NCT03529110	75.8% vs 34.1% 12-month PFS	0.28 [43]
Trastuzumab hyaluronidase^a^	HER2+	–	–	- [44]
Pertuzumab/trastuzumab & hyaluronidase^a^	HER2+	–	–	- [45]
Margetuximab^b^	HER2+	–	–	- [46]
Tucatinib	HER2+	HER2CLIMB, NCT02614794	81 months PFS	0.54 [47]
Sacituzumab govitecan	TNBC	ASCENT, NCT02574455	19 months PFS	0.43 [48]
Pembrolizumab	TNBC	KEYNOTE-355, NCT02819518	4.1 months PFS	0.65 [49]
Elacestrant	HR+	EMERALD, NCT03778931	1.9 months PFS	0.55 [50]
Capivasertib	HR+	CAPItello-291, NCT04305496	6 months PFS	0.50 [51]

HER2/HER2-positive breast cancers (HER2+), hormone receptor-positive breast cancers (HR+), hereditary breast cancers driven by germline mutations in *BRCA* genes 1 or 2 (*gmBRCA*), triple negative breast cancers (TNBC), progression-free survival (PFS), overall survival (OS), invasive disease-free survival (iDFS).

a Trials compared adverse reactions in subcutaneous formulations (with hyaluronidase) versus previously approved intravenous formulations, not survival benefits.

b Trial compared adverse reactions in margetuximab versus trastuzumab (previously approved), not survival benefits.

The 19 targeted therapies FDA approved to treat breast cancer between 2012 and 2023 have had an overall positive effect on breast cancer outcomes, including increased survival and improved quality of life [27]. The results of this evaluation underscore the important role Komen funded research has played in the development of these drugs through both direct investment in pivotal research studies and investment in the investigators who contributed to pivotal studies that supported FDA approval for clinical use. More broadly, it highlights the importance of patient-centered, nonprofit organization support of basic research that is foundational to the development of cancer therapies and ongoing support for investigators working in collaboration to test new therapies in clinical trials.

Komen’s greatest direct research funding impact was for basic research that supported the development of 11 of the 19 approved drugs. These studies were pivotal in identifying and characterizing drug targets, a necessary first step in the drug development pipeline. Examples of Komen’s research funding impact on drug development include: (1) PI3K/mTOR/Akt inhibitor drugs used to treat hormone receptor-positive breast cancers [28], the most commonly diagnosed type of breast cancer accounting for 70%–80% of the estimated 310,720 newly diagnosed invasive breast cancers in women in the United States in 2024 [21]; (2) PARP inhibitors used to treat hereditary breast cancers in people with germline mutations in *BRCA* genes that dramatically increase the risk for developing aggressive breast cancers relative to people without these mutations [29]; and (3) HER2 kinase inhibitors used to treat HER2-positive breast cancers. These results demonstrate the impact of Komen’s funding in basic research that contributed to drug development.

Komen’s research funding support for investigators conducting and contributing to clinical trial studies also played an important role in the path to FDA approval. Funding for scientific research primarily supports the scientists who conduct research [30]. Research funds to support key personnel and staff salaries as well as materials for research not only allows scientists to complete the aims of funded studies; it also allows researchers time and bandwidth to apply their expertise to collaborative studies that contribute to advances in clinical care for diseases like breast cancer, including the development of drugs. Research funding also enables laboratories to train staff, graduate students, postdoctoral fellows, and medical fellows who then go on to contribute to development of research outcomes like new drugs. Komen’s investigator touchpoints for all 19 breast cancer drugs approved by the FDA between 2012 and 2023 measure these vital contributions to the drug development pipeline. For example, an investigator who was supported by Komen funding as a postdoctoral fellow and later as an early career investigator went on to contribute to pivotal clinical trial studies that led to FDA approval of 2 different drugs in our analysis [31,32].

Identifying points of impact for both direct funding for research studies and ongoing funding support for investigators conducting research across the pipeline of drug development is an innovative method to assess the full scope of Komen’s contribution to the development of drugs that advance breast cancer clinical care. Komen directly funded basic research studies that identified drug targets, demonstrated their clinical relevance, and revealed the mechanisms through which they drive breast cancer tumorigenesis and disease progression. Komen’s ongoing support for investigators in the field of breast cancer research is equally important, as it fosters retention in the field, collaborative research that contributes to clinical trial testing of new therapies, and training for the next generation of researchers who go on to contribute to these pivotal studies.

While innovative, limitations in this evaluation include literature bias, meaning citations within pivotal published studies are often heavily skewed in favor of collaborators and in-network colleagues. Our criteria for classifying a study as “pivotal” was based on bibliometric linkage from citations traced back from published Phase III clinical trial results. In addition, we worked with Komen’s Scientific Advisory Board, recognized leaders in the field of breast cancer research who have extensive knowledge of these targeted therapy drugs, to develop this novel evaluation methodology. We chose this methodology rather than surveying Komen grantees to identify pivotal studies to avoid bias in our analyses. While grantees reported information on contributions to some of these drugs, we did not use self-reported contributions in our data collection. These reports were used for data validation purposes but did not change our methodology. This approach, however, may have missed some important studies, and experts in the field may disagree with our designation and inclusion/exclusion of particular studies. Our analysis relied heavily on data collected and curated in Komen databases, including grant progress reports and publications produced by Komen-funded grants as noted by funded investigators. There were some gaps in progress report publication data collection for Komen’s databases from 1982 to the early 2000s. Also, we found gaps in acknowledgement of Komen funding for several studies that were likely Komen-funded. Thus, it is possible that our evaluation did not capture all Komen touchpoints across the pipeline for each drug.

Multiple funders typically contribute to successful drug development, and this evaluation did not assess the contributions of others, such as the NIH or other nonprofits, to the development of the identified drugs. To our knowledge, other funders have not reported similar evaluations of their research funding impact on the discovery and development of these drugs using this methodology for comparison, and access to external verified funding sources is limited. However, throughout the evaluation, we noted frequent citation of federal and other non-profit sources of funding in pivotal published studies. This is not unexpected, as translational research requires financial support from multiple sources, and we acknowledge that other funders contributed to pivotal studies in addition to Komen.

Despite these limitations, our analysis demonstrates Komen’s broad research funding impact for the development of all 19 targeted therapy drugs FDA approved to treat breast cancer between 2012 and 2023. We show that Komen, a patient-centered nonprofit organization, successfully funded research that expands and improves breast cancer clinical care for all major subtypes and stages of breast cancer, improving clinical care and outcomes for people diagnosed with breast cancer. We will continue to evaluate the impact of Komen’s research funding investments by refining data collection processes, leveraging ongoing and retrospective product tracking for Komen funded research, and using innovative research impact assessment tools [10]. Future analyses may include impact assessments of Komen’s research funding on changes to standard of care for breast cancer including new combinations of drugs, predictive biomarkers developed as part of Komen’s commitment to advancing precision medicine [33], and ongoing studies testing the safety of treatment de-escalation to avoid overtreatment and reduce toxic side effects.

Komen remains a catalyst in nonprofit funding of research that is critical to drug development and advancing breast cancer care. We will continue to assess the impact of our research support to ensure investments ultimately benefit patients and improve outcomes for all people with a breast cancer diagnosis.
